# Characteristics of acute kidney injury and its impact on outcome in patients with acute-on-chronic liver failure

**DOI:** 10.1186/s12876-022-02316-8

**Published:** 2022-05-11

**Authors:** Yue Huang, Junjun Cai, Fushuang Ha, Beichen Guo, Shaojie Xin, Zhongping Duan, Tao Han

**Affiliations:** 1 Department of Hepatology and Gastroenterology, The Third Central Clinical College of Tianjin Medical University, Tianjin, 300170 China; 2 Department of Hepatology and Gastroenterology, Tianjin Union Medical Center affiliated to Nankai University, 190 Jieyuan Road, Hongqiao District, Tianjin, 300121 China; 3 Department of Hepatology and Gastroenterology, The Third Central Hospital of Tianjin, 83 Jintang Road, Hedong District, Tianjin, 300170 China; 4 Artificial Cell Engineering Technology Research Center, Tianjin, China; 5 Tianjin Key Laboratory of Extracorporeal Life Support for Critical Diseases, Tianjin, China; 6 Liver Failure Treatment and Research Center, The Fifth Medical Center of Chinese, PLA General Hospital, Beijing, China; 7 Liver Disease Center (Difficult and Complicated Liver Diseases and Artificial Liver Center), Beijing You’an Hospital Affiliated to Capital Medical University, Beijing, China

**Keywords:** Liver failure, Acute kidney injury, Prognosis, Nomogram

## Abstract

**Objective:**

Acute kidney injury (AKI) is a common and life-threatening complication of liver failure. The purpose of this study is to construct a nomogram and online calculator to predict the development of hospital-acquired acute kidney injury (HA-AKI) in patients with acute-on-chronic liver failure (ACLF), which may contribute to the prognosis of ACLF.

**Methods:**

574 ACLF patients were evaluated retrospectively. AKI was defined by criteria proposed by International Club of Ascites (ICA) and divided into community-acquired and hospital-acquired AKI (CA-AKI and HA-AKI). The difference between CA-AKI and HA-AKI, factors associated with development into and recovered from AKI periods. The risk factors were identified and nomograms were developed to predict the morbidity of HA-AKI in patients with ACLF.

**Results:**

Among 574 patients, 217(37.8%) patients had AKI, CA-AKI and HA-AKI were 56 (25.8%) and 161 (74.2%) respectively. The multivariate logistic regression model (KP-AKI) for predicting the occurrence of HA-AKI were age, gastrointestinal bleeding, bacterial infections, albumin, total bilirubin, blood urea nitrogen and prothrombin time. The AUROC of the KP-AKI in internal and external validations were 0.747 and 0.759, respectively. Among 217 AKI patients, 81(37.3%), 96(44.2%) and 40(18.4%) patients were with ICA-AKI stage progression, regression and fluctuated in-situ, respectively. The 90-day mortality of patients with AKI was 55.3% higher than non-AKI patients 21.6%. The 90-day mortality of patients with progression of AKI was 88.9%, followed by patients with fluctuated in-situ 40% and regression of AKI 33.3%.

**Conclusions:**

The nomogram constructed by KP-AKI can be conveniently and accurately in predicting the development of HA-AKI, and AKI can increase the 90-day mortality significantly in ACLF patients.

*Trial registration* Chinese clinical trials registry: ChiCTR1900021539.

**Supplementary Information:**

The online version contains supplementary material available at 10.1186/s12876-022-02316-8.

## Introduction

Acute-on-chronic liver failure (ACLF) is an acute deterioration of liver function within a short period under acute precipitating insult, which manifests as multiple organ failure and high 28 and 90-day mortality [1, 2]. Acute kidney injury (AKI), which is the most common complication of ACLF, is characterized by a sudden decline in renal function [3]. Patients with liver disease are prone to intravascular volume depletion secondary to gastro-intestinal bleeding, and tend to be susceptible to usage of diuretic and aminoglycosides. Most importantly, because of the hyperdynamic circulatory state, patients with cirrhosis are highly susceptible to renal events associated with a further decrease in effective arterial blood volume [4].

A previous study showed that among 1032 patients with ACLF who had underlying cirrhosis, 11.7% had AKI at admission (community-acquired), and 30.9% developed AKI during hospitalization (hospital-acquired) [5]. AKI is an early-stage disease, and exacerbation of the initial kidney injury can eventually progress to irreversible damage to kidney function [6, 7]. Approximately 2/3 of AKI episodes in patients with cirrhosis are functional or volume-responsive and reversible [8]. However, patients with even mild renal impairment (peak AKI stage 1) had significantly higher 90-day mortality than those without any renal impairment [4, 9]. Moreover, patients who completely recovered renal function at the end of AKI episodes also had a much higher 90-day mortality than those who had never suffered from AKI [10]. However, knowledge of the characteristics of AKI and identifying the main determinants of the occurrence of HA-AKI of patients with ACLF is limited.

This study aimed to investigate the characteristics of AKI in ACLF patients. We compared community-acquired and hospital-acquired AKI (CA-AKI and HA-AKI, respectively) patients and evaluated the risk factors associated with development and recovery from AKI. Furthermore, we constructed a visual nomogram and online calculator for predicting the HA-AKI based on a model which may be used by physicians to determine the occurrence of AKI and to minimize poor prognosis.

## Patients and methods

### Study participants and data collection

**Fig. 1:**
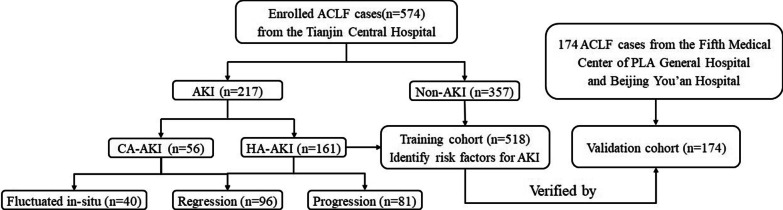
A flow chart explaining the patient’s selection process

The data of 574 patients with ACLF admitted to Tianjin Third Central Hospital between June 2006 and May 2019, 174 patients with ACLF from the Fifth Medical Center of PLA General Hospital (n = 82) and Beijing You’an Hospital (n = 92) were analyzed retrospectively (Fig. 1). All data (clinical data and scoring models: CTP, MELD and MELD-Na) were collected or calculated from the electronic medical records of patients. (The estimated glomerular filtration rate (eGFR) was derived from MDRD equation [11]).

Patients who were discharged alive from hospital were followed at least 3 months by telephone. Exclusion criteria were as follows: age < 18 or age > 70 years old, with hepatic and non-hepatic neoplasia, chronic kidney disease under hemodialysis treatment before admission, previous kidney or liver transplant, clinically estimated life expectancy < 3 days.

This retrospective study was approved by the Ethics Committee of Tianjin Third Central Hospital, Beijing You’an Hospital Affiliated to Capital Medical University and the Fifth Medical Center of PLA General Hospital and conducted according to the principles of the Declaration of Helsinki (approved No. of ethic committee: SZX-IRB-SOP-016(F)-002-01). This trial was registered in the Chinese clinical trials registry: ChiCTR1900021539. We obtained written informed consent from all patients or their legal guardian.

ACLF was defined according to the APAPL criteria: total bilirubin (TBIL) ≥ 5 mg/dL or ≥ 85umol/L and international normalized ratio of prothrombin time (INR) ≥ 1.5 or prothrombin activity (PTA) ≤ 40%, complicated with ascites and/or hepatic encephalopathy noted within 4 weeks in a patient with previously chronic liver disease [12]. The diagnosis of cirrhosis was based on previous liver-biopsy findings or a composite of clinical signs and findings provided by laboratory tests, endoscopy and radiologic imaging. Hepatic encephalopathy (HE) was defined and graded by the West Haven criteria. Definitions of bacterial infections were depicted as well as in reference 13 [13]. The definition of AKI is an absolute increase in serum creatinine (sCr) of ≥ 0.3 mg/dL from baseline within 48 h or a percent increase of sCr ≥ 50% from baseline within the prior 7 days and then classified it into stage 1, 2, and 3 [3]. The baseline sCr value is the value closest to admission time to the hospital within the previous 3 months. When sCr measurement has never been done, the sCr on admission was used as baseline [3]. Community-acquired AKI were patients diagnosed with AKI on admission. Hospital-acquired AKI were patients which without AKI on admission and developed AKI during the hospitalization [14]. AKI linked to cirrhosis may assume a prerenal form, hepatorenal syndrome (HRS) or acute tubular necrosis (ATN) [15]. While the define of pre-renal azotemia is recovery of renal function (decrease in serum creatinine) after correction of hemodynamic abnormalities [16]. The define of ATN is all cases of AKI preceded by septic or posthemorrhagic shock, prolonged dehydration, severe pancreatitis, exposure to nephrotoxic substances (aminoglycosides, contrast agents) or major surgical interventions [15]. HRS defined by a doubling of the initial sCr to a level greater than 2.5 mg/dL or a 50% reduction of the initial 24-h creatinine clearance to a level lower than 20 mL/min in less than 2 weeks [3]. Progression of AKI was considered as ICA-AKI stage progressed to a higher stage and/or need for renal replacement therapy (RRT). Regression of AKI was considered as ICA-AKI stage regressed to a lower stage. Fluctuated in-situ of AKI was considered as AKI stage neither progression nor regression during hospitalization.

### Statistical analysis

Continuous variables were described by mean ± SD, while categorical variables were expressed by frequency (percentage). Differences between groups continuous variables were compared with Mann–Whitney test or two-tailed t test, categorical variables were compared with fisher exact test or chi-square test. Identify the independent factors and construct the predictive model by multivariate logistic regression. The model was evaluated by the Hosmer–Lemeshow test to appraise the goodness of fit and visualized by nomogram through package “rms” and “ggplot2” in R. Harrells concordance index (C-index), Somer’s D, R^2^, the area under the Receiver Operator Characteristic (AUROC) curve and calibration curves were used as metric to quantify the nomogram performance and accuracy in training and validation cohort.

Finally, Kaplan–Meier survival curves were used to discriminate the relationship between the model and 90-day survival probability through packed “survival” and “survminer” in R and assessed by log-rank test. All data were analyzed by SPSS.24 and R software (version 4.0.5).

## Results

### Baseline characteristics of eligible patients

Among the 574 patients, the mean age was 51.1 ± 12.2, and 434 (75.6%) were male. The most common etiology of liver disease was hepatitis B virus (HBV) infection (51.7%). 27 (4.7%) patients received ICU and RRT during hospitalization and 9(1.5%) patients received liver transplantation in the follow-up. The complications of ACLF on admission were as follows: hepatic encephalopathy (n = 54), ascites (n = 300), GI bleeding (n = 114), bacterial infections (n = 100), and AKI (n = 217). Compared with non-AKI patients, patients with AKI tended to be older and more frequently had GI bleeding and bacterial infections, higher heart rate, WBC count, total bilirubin (TBIL), INR, blood urea nitrogen (BUN), admission sCr, serum potassium, CTP, MELD, and MELD-Na scores, and lower albumin, eGFR and serum sodium values (*P* < 0.05) (Table 1).

**Table 1:** Comparison of characteristics between patients with and without AKI, CA-AKI and HA-AKI

Variables	Non-AKI N = 357	AKI N = 217	*P* value	AKI		
				CA-AKI N = 56	HA-AKI N = 161	*P* value
*Age (years)*	49.6 ± 11.9	53.6 ± 12.1	** < 0.001**	54.1 ± 11.8	53.5 ± 12.2	0.728
*Male-n (%)*	265 (74.2)	169 (77.9)	0.323	45 (80.4)	124 (77.0)	0.604
*Death-n(%)*	77 (21.6)	120 (55.3)	** < 0.001**	33 (58.9)	87 (54.0)	0.0526
Hypertension-n(%)	42 (11.8)	41 (18.9)	**0.019**	10 (17.9)	31 (19.3)	0.818
Diabetes mellitus-n(%)	56 (9.8)	43 (19.8)	0.204	10 (17.9)	33 (20.5)	0.669
*Baseline Cr* (μmol/L)	60.5 ± 18.4	84.9 ± 49.1	** < 0.001**	128.2 ± 70.8	69.9 ± 25.5	** < 0.001**
*Baseline eGFR* (ml/min/1.73m^2^)*	134.6 ± 43.1	109.5 ± 61.1	** < 0.001**	73.4 ± 45.6	122.0 ± 60.9	** < 0.001**
*Peak Cr* (μmol/L)	–	214.2 ± 139.9		276.4 ± 158.8	192.6 ± 126.1	** < 0.001**
*Etiology of liver disease-n (%)*			**0.013**			0.793
Hepatitis B	195 (54.6)	102 (47.0)		25 (44.6)	77 (47.8)	
Alcohol	92 (25.8)	81 (37.3)		23 (41.1)	58 (36.0)	
Other causes	70 (19.6)	34 (15.7)		8 (14.3)	26 (16.1)	
*Complications at admission-n (%)*						
Ascites	182 (51.0)	118 (54.4)	0.429	30 (53.6)	88 (54.7)	0.888
HE	32 (9.0)	22 (10.1)	0.640	7 (12.5)	15 (9.3)	0.497
GI bleeding	51 (14.3)	63 (29.0)	** < 0.001**	19 (33.9)	44 (27.3)	0.349
Bacterial infection	46 (12.9)	54 (24.9)	** < 0.001**	11 (19.6)	43 (26.7)	0.292
*Admission parameters*						
MAP (mmHg)	90 ± 11.5	88.7 ± 14.9	0.117	82.8 ± 15.0	90.7 ± 14.4	** < 0.001**
Heart rate (bpm)	82.6 ± 14	87.2 ± 14.9	** < 0.001**	91.2 ± 18	85.8 ± 13.5	**0.010**
WBC (× 10^9^/L)	7.0 ± 4.4	9.8 ± 6.5	** < 0.001**	12.2 ± 7.2	9.0 ± 6.1	** < 0.001**
PLT (× 10^9^/L)	99.3 ± 62.3	104.3 ± 69.6	0.186	111.4 ± 77.5	101.9 ± 66.7	0.190
ALB (g/L)	29.4 ± 5.1	27.3 ± 5.1	** < 0.001**	26.6 ± 4.5	27.5 ± 5.3	0.140
TBIL (μmol/L)	233.1 ± 129.3	263.1 ± 156.7	**0.009**	261.3 ± 154.4	263.7 ± 157.9	0.462
INR	2.3 ± 0.9	2.4 ± 1.1	**0.034**	2.6 ± 1.4	2.4 ± 1.0	0.077
PT (s)	33.8 ± 10.9	35.7 ± 13.3	**0.033**	36.1 ± 13.2	35.5 ± 13.3	0.391
BUN (mmol/L)	5.3 ± 3.1	10.4 ± 7.3	** < 0.001**	17.7 ± 7.9	7.9 ± 5.1	** < 0.001**
sCr (μmol/L)	60.6 ± 18.4	110.8 ± 80.7	** < 0.001**	213.1 ± 91.7	75.2 ± 31.1	** < 0.001**
Serum Na^+^ (mmol/L)	134.3 ± 5.8	132.3 ± 6.1	** < 0.001**	130.9 ± 7.2	132.8 ± 5.6	0.021
Serum K^+^ (mmol/L)	3.8 ± 0.6	4.0 ± 0.7	**0.004**	4.1 ± 0.9	3.9 ± 0.6	0.029
eGFR (ml/min/1.73m^2^)*	134.6 ± 43.1	105.2 ± 62.9	** < 0.001**	62.2 ± 45.7	120.2 ± 61.2	** < 0.001**
CTP	11.1 ± 1.7	11.5 ± 1.8	**0.006**	11.8 ± 1.7	11.4 ± 1.8	0.118
MELD	18.2 ± 5.7	22.6 ± 8.5	** < 0.001**	30.4 ± 7.1	20.0 ± 7.2	** < 0.001**
MELD-Na	22.5 ± 8.5	29.3 ± 10.9	** < 0.001**	38.9 ± 9.8	26.0 ± 9.2	** < 0.001**
*Initial ICA-AKI stage-n*						
Stage 1/2/3		132/58/27		30/14/12	102/44/15	0.060
*Peak ICA-AKI stage-n*						
Stage 1/2/3		84/60/73		25/9/22	59/51/51	0.080

*ALB* albumin, *BUN* blood urea nitrogen, *CTP* child-turcotte-pugh, *eGFR* estimated glomerular filtration rate, *HE* hepatic encephalopathy, *INR* international normalized ratio, *MAP* mean arterial pressure, *MELD* model for end-stage liver disease, *PLT* platelet, *sCr* serum creatinine, *TBIL* total bilirubin, *WBC* white blood cells

^*^Estimated by MDRD

Bold represented *P* < 0.05 with statistically significant

Among 217 patients with AKI, 132 (60.8%), 58 (26.7%), and 27 (12.4%) patients met ICA-AKI stages 1, 2, and 3, respectively. 56 (25.8%) were community-acquired and 161 (74.2%) were hospital-acquired. Patients with CA-AKI had a higher heart rate, baseline sCr, peak sCr, admission sCr, WBC counts, BUN, serum potassium values, MELD and MELD-Na scores, and lower MAP, serum sodium, and baseline eGFR values compared with the HA-AKI group (*P* < 0.05). There was no statistical significance in others. (Table 1).

### Risk factors and nomogram for HA-AKI in ACLF

**Fig. 2:**
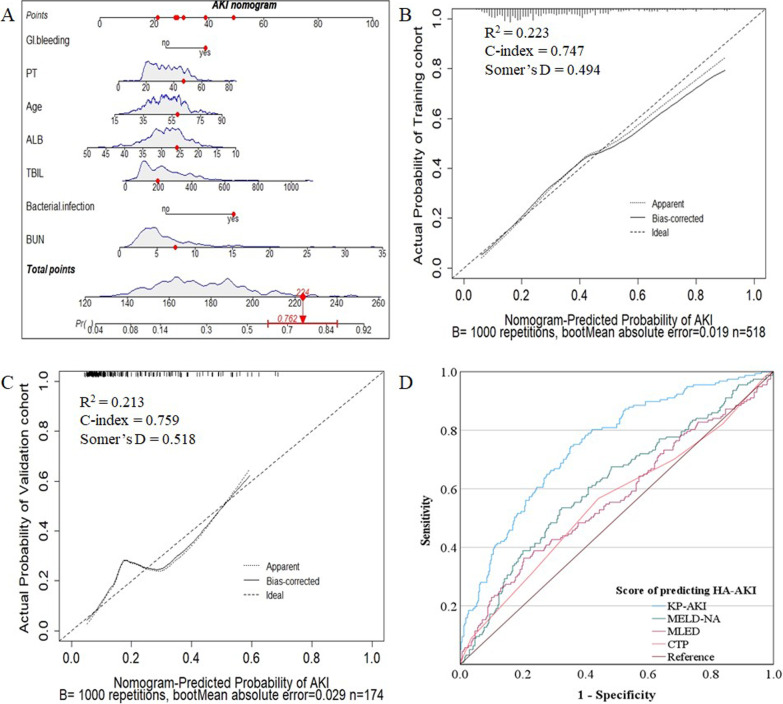
**A** The nomogram constructed by KP-AKI model in training cohort. As shown in the **A**, a 59-year-old patient upon admission with a bacterial infection and gastrointestinal bleeding, the BUN was 7.39 mmol/L and TBIL was 193.1 μmol/L, PT was 47 s and ALB was 25.8 g/L, the total point added up to 224, which represents the occurrence of HA-AKI was 76.2%. **B** Calibration curves in the internal training cohort. **C** Calibration curves in the external validation cohort. **D** ROC curves for several scoring systems in identified for the development of AKI

Removing 56 patients had community-acquired AKI on admission, the remaining 518 patients were used as the training cohort to constructed the KP-AKI model by multivariate logistic regression for predicting the HA-AKI (Table 2) and visualized as a nomogram (Fig. 2A). Risk factors as follow: age (*P*, OR, 95%CI) (0.009, 1.023, 1.006–1.041), GI bleeding (0.015, 1.892, 1.131–3.166), bacterial infection (< 0.001, 2.967, 1.751–5.027), ALB (0.010, 0.942, 0.901–0.986), TBIL (0.001, 1.003, 1.001–1.004), BUN (< 0.001, 1.128, 1.067–1.193), and PTs (0.015, 1.022, 1.004–1.041) (Table 2). KP-AKI model was: 0.023 × Age + 0.638 × GI bleeding (1 if GI bleeding, 0 otherwise) + 1.087 × bacterial infection (1 if bacterial infection, 0 otherwise) − 0.060 × ALB (g/L) + 0.003 × TBIL (μmol/L) + 0.121 × BUN (mmol/L) + 0.022 × PT(s) − 2.828. [Hosmer–Lemeshow test (χ^2^ = 11.042, *P* = 0.199) and Omnibus test (χ^2^ = 89.203, *P* < 0.001)]. Online at https://tyhyue12.shinyapps.io/APP-Nomapp/.

**Table 2:** Risk factors for HA-AKI in patients with ACLF

Variables	Estimate	OR (95%CI)	Standard error	Wald X^2^	*P* value
Age (years)	0.023	1.023 (1.006–1.041)	0.009	6.746	0.009
GI bleeding	0.638	1.892 (1.131–3.166)	0.263	5.901	0.015
Bacterial infection	1.087	2.967 (1.751–5.027)	0.269	16.333	< 0.001
TBIL (μmol/L)	0.003	1.003 (1.001–1.004)	0.001	11.591	0.001
BUN (mmol/L)	0.121	1.128 (1.067–1.193)	0.028	18.080	< 0.001
ALB (g/L)	− 0.060	0.942 (0.901–0.986)	0.023	6.716	0.010
PT (s)	0.022	1.022 (1.004–1.041)	0.009	5.956	0.015

If GI bleeding or bacterial infection present 1, otherwise 0

174 patients with ACLF from the Fifth Medical Center of PLA General Hospital and Beijing You’ an Hospital perform external verification as a validation group (Fig. 1, Additional file 1: Digital Content 1). In the training cohort and validation cohort, the C-index was 0.747 and 0.759, the Somer’s D was 0.494 and 0.518, the R^2^ was 0.223 and 0.213. The trends of the calibration curves of the internal training cohort (mean absolute error = 0.019) and external validation cohort (mean absolute error = 0.029) were similar (Fig. 2B, C).

The AUROC of the KP-AKI model (AUC, 95%CI, *P*) (0.747, 0.702–0.792, < 0.001) was the highest, followed by MELD-Na (0.612, 0.558–0.666, < 0.001), MELD (0.567, 0.511–0.624, 0.016), and CTP (0.554, 0.497–0.610, 0.055) for predicting the development of HA-AKI (Fig. 2D).

### Comparison among patients with AKI stage progression, regression and fluctuated in-situ

Among the 217 AKI patients, 81 (37.3%) progressed to a higher AKI stage, 96 (44.2%) regressed to a lower AKI stage, and 40 (18.4%) patients fluctuated in situ. At discharge from the hospital, 84 (38.7%), 60 (27.6%), and 73 (33.6%) patients had reached peak stage 1, 2, and 3 ICA-AKI, respectively. Patients with progression of AKI tended to have an older age, higher mortality, more presence HBV infection, encephalopathy, hepatorenal syndrome (HRS) and acute tubular necrosis (ATN), higher baseline and peak sCr values, higher value of TBIL, INR and BUN. higher CTP, MELD and MELD-Na scores at the time for diagnosis of AKI than patients without progression of AKI (*P* < 0.05). (Table 3). The independent factors associated with the progression of AKI by multivariate logistic regression analysis were HA-AKI, alcohol liver disease, BUN, INR, baseline eGFR, presence of PRA and ATN (Table 4).

**Table 3:** Comparisons among patients with AKI progression, regression and fluctuated in-situ

Variables	Fluctuated in-situ N = 40	Regression N = 96	Progression	*P* value
			N = 81	
*Age (years)*	55.5 ± 9.1	50.6 ± 12.1	56.4 ± 12.7	**0.003**
*Male-n (%)*	28 (70.0)	81 (84.4)	60 (74.1)	0.107
*Death-n (%)*	16 (40.0)	32 (33.3)	72 (88.9)	** < 0.001**
MAP (mmHg)	90.6 ± 11.9	86.1 ± 17.3	90.8 ± 12.7	0.074
Heart rate (bpm)	88.4 ± 15.9	89.0 ± 16.1	84.5 ± 12.6	0.115
*Baseline Cr* (μmol/L)	78.7 ± 39.3	78.4 ± 33.0	95.8 ± 65.4	**0.041**
*Baseline eGFR* (ml/min/1.73m^2^)	110.3 ± 53.5	111.2 ± 47.6	107.1 ± 77.3	0.903
*Peak Cr (μmol/L)*	147.5 ± 82.1	173.6 ± 112.1	295.3 ± 154.1	** < 0.001**
*CA-AKI/HA-AKI-n*	11/29	28/68	17/64	0.447
*Initial ICA-AKI stage-n*				
Stage 1/2/3	28/10/2	58/23/15	46/25/10	0.383
*Peak ICA-AKI stage-n*				
Stage 1/2/3	26/12/2	49/29/18	9/19/53	** < 0.001**
*Type of AKI-n(%)*				** < 0.001**
ATN	2 (5.0)	15 (15.6)	56 (32.1)	
PRA	22 (55.0)	77 (80.2)	8 (9.9)	
HRS	16 (40.0)	15 (15.6)	47 (58.0)	
*Etiology of liver disease- n (%)*				** < 0.001**
Hepatitis B	19 (47.5)	35 (36.5)	48 (59.3)	
Alcohol	10 (25.0)	50 (52.1)	51 (25.9)	
Other causes	11 (27.5)	11 (11.5)	12 (14.8)	
*Complications during hospitalization-n (%)*				
Ascites	26 (65.0)	70 (72.9)	58 (71.6)	0.643
HE	15 (35.0)	19 (19.8)	33 (40.7)	**0.008**
GI bleeding	9 (22.5)	22 (22.9)	19 (23.5)	0.992
Bacterial infection	30 (75.0)	74 (77.1)	70 (86.4)	0.198
Shock	2 (5.0)	10 (10.4)	13 (18.0)	0.182
Mechanical ventilation	0 (0.0)	3 (3.1)	8 (9.9)	**0.034**
*Parameters at diagnosis of AKI*				
WBC (× 10^9^/L)	9.6 ± 6.6	11.2 ± 7.1	11.9 ± 7.0	0.219
PLT (× 10^9^/L)	85.4 ± 66.0	100.4 ± 63.6	89.9 ± 67.4	0.383
ALB (g/L)	29.3 ± 5.1	28.0 ± 4.6	27.5 ± 4.8	0.148
TBIL (μmol/L)	270.9 ± 185.1	242.4 ± 146.9	308.2 ± 168	**0.030**
INR	2.2 ± 1.1	2.4 ± 1.0	3.0 ± 2.0	**0.005**
PT (s)	29.2 ± 13.4	28.0 ± 9.5	31.2 ± 14.7	0.237
BUN (mmol/L)	11.3 ± 5.7	13.9 ± 9.6	16.5 ± 7.7	**0.004**
sCr (μmol/L)	135.6 ± 77.7	157.3 ± 84.7	183.1 ± 86.9	**0.011**
Serum Na^+^ (mmol/L)	132.4 ± 4.8	132.3 ± 6.4	130.1 ± 7.3	**0.045**
Serum K^+^ (mmol/L)	4.1 ± 0.7	3.9 ± 0.7	5.3 ± 10.3	0.316
CTP	11.0 ± 1.5	11.8 ± 1.4	12.3 ± 1.7	** < 0.001**
MELD	24.7 ± 7.0	25.7 ± 7.9	31.5 ± 8.4	** < 0.001**
MELD-Na	30.3 ± 10.8	32.0 ± 11.7	40.7 ± 13.6	** < 0.001**

Bold represented *P* < 0.05 with statistically significant

**Table 4:** Predictors for progression and regression of AKI, respectively

Variables	Estimate	OR (95%CI)	Standard error	Wald X^2^	*P* value
*Predictors for progression*					
HA-AKI	1.459	4.301 (1.599–11.57)	0.505	8.350	0.004
Alcohol abuse	− 1.246	0.288 (0.116–0.716)	0.465	7.182	0.007
BUN	0.057	1.058 (1.012–1.107)	0.023	6.259	0.012
INR	0.381	1.463 (1.092–1.96)	0.149	6.519	0.011
PRA	− 3.666	0.026 (0.009–0.077)	0.560	42.848	< 0.001
ATN	1.751	5.763 (1.724–19.263)	0.616	8.093	0.004
Baseline eGFR	0.011	1.011 (1.003–1.019)	0.004	6.869	0.009
AUC 0.918 (0.892–0.953)					
*Predictors for regression*					
HA-AKI	− 0.996	0.369 (0.145–0.943)	0.478	4.335	0.037
Alcohol abuse	1.464	4.324 (2.04–9.168)	0.383	14.585	< 0.001
PRA	3.500	33.100 (12.661–86.538)	0.490	50.937	< 0.001
Baseline eGFR	− 0.010	0.990 (0.982–0.998)	0.004	5.561	0.018
AUC 0.864 (0.817–0.910)					

### Impact of AKI on 90-day survival in ACLF patients

**Fig. 3:**
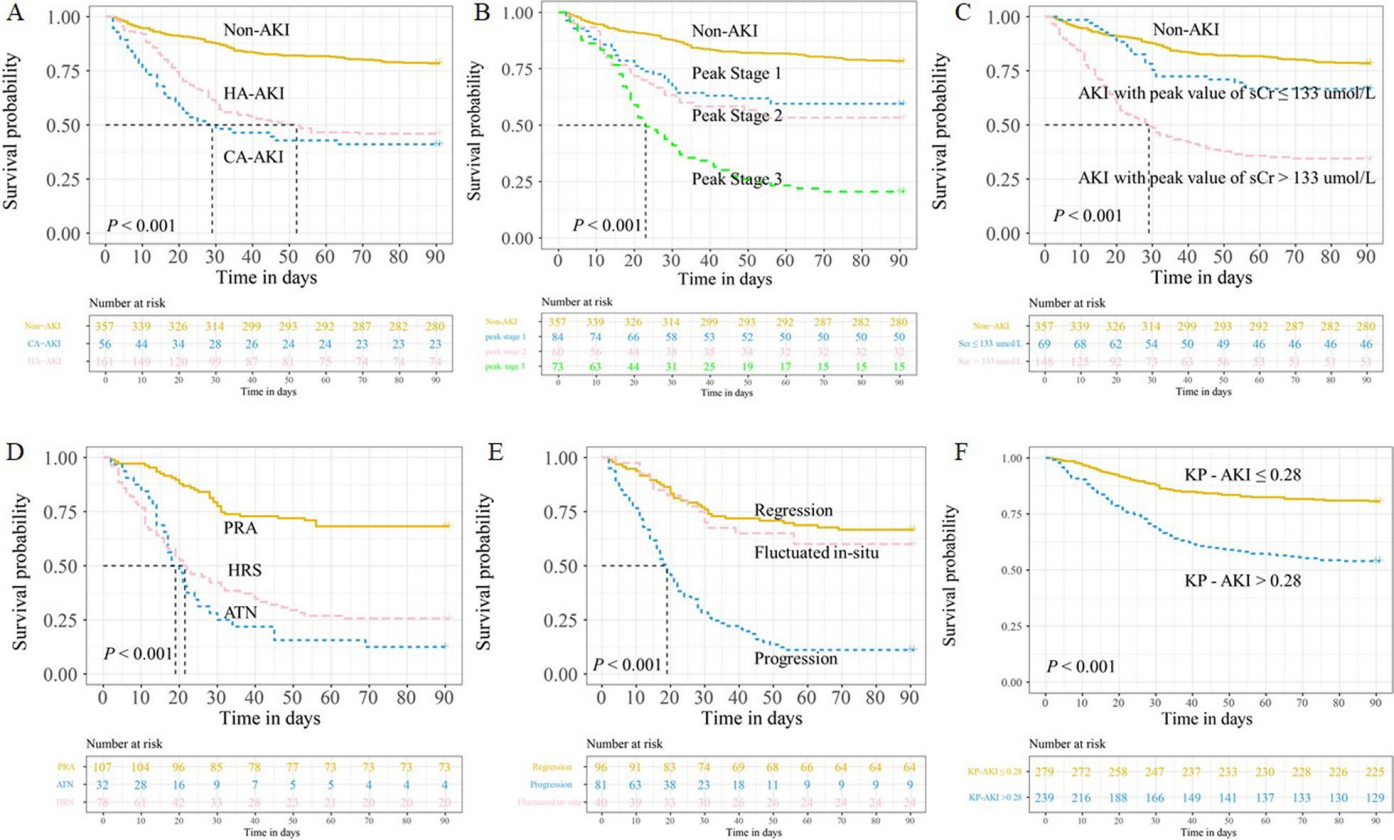
Kaplan–Meier survival analyses of the 90-day mortality in ACLF patients. **A** Patients without AKI, with CA-AKI and HA-AKI. **B** Patients without AKI and with ICA-AKI stage 1, 2 and 3. **C** Patient without AKI, with peak sCr < 133 μmol/L and with peak sCr ≥ 133 μmol/L. **D** Patient with ATN, HRS and PRA, respectively. **E** Patient with progression of AKI, regression of AKI and fluctuated in-situ. **F** Patients divided by cut-off value of KP-AKI: High group (KP-AKI score ≥ 0.28) and Low group (KP-AKI score < 0.28)

The incidence of 90-day mortality in patients with AKI was 54.8% (CA-AKI, 58.9%; HA-AKI, 54.0%), which was higher than that in patients without AKI (21.6%) (Fig. 3A). The 90-day mortality rates of the patients with peak ICA-AKI stages 1, 2, and 3 were 40.4%, 46.6%, and 79.5%, respectively. Mortality increased in a stage-dependent manner with AKI severity (Fig. 3B). The 90-day mortality of AKI patients with a peak sCr ≥ 133 µmol/L (65.5%) was significantly higher than that of patients with a peak sCr < 133 µmol/L (33.3%) (Fig. 3C). Furthermore, we noted a strong relationship between AKI type and mortality. Patients with acute tubular necrosis had the worst prognosis than those with HRS and pre-renal azotemia (PRA) (Fig. 3D). The 90-day mortality of patients with progression of AKI was 88.9%, followed by patients with fluctuation in situ (40%) and regression (33.3%) (Fig. 3E). We also investigated the relationship between the KP-AKI model and 90-day mortality in ACLF patients. These patients were further classified into two groups using the cut-off value of the KP-AKI model score: a high group (KP-AKI score ≥ 0.28) and a low group (KP-AKI score < 0.28). The 90-day mortality rate in the high KP-AKI group was higher (46.0%) than that in the low KP-AKI group (19.4%) (*P* < 0.001) (Fig. 3F).

## Discussion

AKI is a common and rapidly progressive in patients with ACLF and is associated with significantly worse outcome. The 30-day mortality of ACLF patients with AKI remains very high (about 50%) [17]. Because of the ominous prognosis and potential reversibility of AKI in ACLF patients, identifying the main determinants of the occurrence of HA-AKI is essential for developing new targeted therapies.

In our study, 37.8% patients had AKI (25.8% CA-AKI and 74.2% HA-AKI). This finding is consistent with the previous study which the prevalence of AKI was 42.6% in patients with ACLF who met CMA criteria with underlying cirrhosis (CA-AKI and HA-AKI accounted for 27.5% and 72.5%, respectively) while no more details of the comparisons between HA-AKI and CA-AKI in study [5]. Another prospective study showed a different result from ours, that is, the overall prevalence of AKI in patients with liver cirrhosis is 35% (CA-AKI and HA-AKI account for 25% and 10% respectively) [14], which may be due to the different basic status of patients included in the study. Patients in our study were ACLF who met APASL criteria, and Patidar KR's study [14] patients were liver cirrhosis. In Patidar KR's study, CA-AKI had a higher AKI stage at the time of AKI diagnosis and peak, and a higher mortality than HA-AKI, which is similar to the trend in our study. Nevertheless, our findings suggest that prompt identification and treatment of AKI affects outcomes, and patients with CA-AKI should be monitored closely after discharge to avoid poor outcomes.

In this study, we found that GI bleeding, bacterial infection, age, ALB, TBIL, BUN, PTs were the independent risk factors for HA-AKI in ACLF patients. The predictive model of KP-AKI encompasses the above seven factors, which are more precise in predicting the incidence of AKI than the conventional scoring systems (CTP, MELD, and MELD-Na scores). Recently, a PRIO model was developed to predict AKI in ACLF patients [17]. However, the primary etiology of chronic liver disease is alcoholic liver disease (ALD). Moreover, the PRIO model does not distinguish between HA-AKI and CA-AKI [17], which means the predicted model includes many cases that have already occurred. Our study focused on the prediction of the occurrence of HA-AKI, which accounts for the majority of AKI cases; on the other hand, the predominant etiology of chronic liver disease is HBV infection.

GI bleeding, bacterial infection as the main risk factors of HA-AKI in our study, may due to circulatory dysfunction and systemic inflammation are the primary pathogeneses of AKI [18]. Patients with liver cirrhosis are particularly susceptible hyperdynamic circulatory state and low arterial blood volume [4]. Presence of GI bleeding, hemorrhagic shock or septic shock aggravates the shortage of effective arterial blood volume, further activating the sympathetic nervous system, renin–angiotensin–aldosterone system, and non-osmotic release of antidiuretic hormone [19]. As the disease progresses, splanchnic and systemic vasodilatations worsen and activate vasoconstrictive systems, leading to renal vasoconstriction. Additionally, insufficient cardiac output further contributes to the diminution of kidney perfusion and progression of kidney injury [19, 20]. Due to the altered gut microflora, loss of intestinal integrity, translocation of bacteria, immune dysfunction and portal systemic shunting on ACLF patients [21], Bacterial infection is a common and fatal complication in patients with ACLF, and can also act as a trigger for AKI and ACLF development and progression [22]. Bacterial infection triggers inflammation through pathogen-associated molecular patterns, resulting in the release of inflammatory mediators that cause tissue damage, which in turn leads to the release of damage-associated molecular patterns, which further drives the inflammatory process. Indeed, serum levels of the pro-inflammatory cytokine interleukin-6 (IL-6), tumor necrosis factor-α (TNF-α), C-reactive protein, and lipopolysaccharide are elevated in patients with cirrhosis in parallel with the severity of disease, and are associated with poor outcomes [23, 24]. An excessive inflammatory response can induce apoptosis/necrosis of renal parenchyma and interstitial cells through various inflammatory pathways, eventually causing kidney dysfunction [18].

Serum bilirubin, albumin, and coagulopathy were the primary parameters that were found to indicate the severity of liver dysfunction. ALB and bilirubin mirrored liver synthesis and hepatocellular injury. Hypoproteinemia and bilirubin levels were related to a high incidence of AKI. High bilirubin can lead to an underestimation of sCr, which can obscure renal insufficiency in clinical situations [25]; Accumulated unbound bilirubin inhibits oxidative phosphorylation, which leads to changes in renal tubular cell permeability and damage renal function [26, 27]. Coagulation function is complicated in ACLF, which is characterized by a precarious balance between bleeding and thrombosis due to repeated hemostasis, organ failure, sepsis, and anticoagulant use in ACLF patients. Studies have shown that hypocoagulable and hypofibrinolytic states were correlated with systemic inflammation, and could contribute to organ failure and higher short-term mortality of ACLF patients [28, 29]. The abnormal coagulation regulating pathway in inflammation can damage renal function through immunothrombosis of the kidney, or coagulation-induced production of mitogenic factors (IL-6, IL-17, IL-22, and miRNAs), which can trigger epithelial cell proliferation [30]. ACLF patients with high level bilirubin, low level albumin abnormal coagulation function were more likely to develop AKI in this study.

It can be seen from the above, the KP-AKI model contains serum bilirubin, ALB, coagulopathy, GI bleeding and bacterial infection which indicates the severity of liver dysfunction and pathophysiology and predictors of AKI in ACLF patients is possible to identify and stratify ACLF patients at risk of AKI and mortality.

In addition, among 217 AKI patients, 81 (37.3%), 96 (44.2%), and 40 (18.4%) patients had ICA-AKI stage progression, regression, and fluctuation in situ, respectively. This rate of progression is much higher than that reported by Fagundes et al. [10]. In a series of hospitalized patients with cirrhosis, which was only 22%. The discrepancy may be due to differences in the severity of disease among the patient populations, or the methods used for the prevention or treatment of AKI. A recent study showed that AKI in ACLF patients is more likely to be associated with structural kidney injury and is more progressive, showing a poorer response to terlipressin treatment and a worse prognosis than that of DC patients [31]. Independent factors associated with progression of AKI were HA-AKI, alcohol liver disease, BUN, INR, baseline eGFR, presence of PRA and ATN, while different from another study which the factors associated with the progression of AKI were HE, chronic kidney impairment, severe liver and circulatory failure, low serum sodium concentration, and high leukocyte count [10]. It is worth noting that the 90-day mortality of patients with both ICA-AKI stage 3 and progression of AKI was the highest. Therefore, close monitoring outside the hospital for early identification, and timely treatment to prevent the progression of AKI is necessary to improve poor prognosis.

Though we patients were HBV etiology preponderance and obviated the influence of CA-AKI, our study also has a number of limitations. First, it is a retrospective study may cause the occurrence of selection bias (retrospective nature) and lack of validation data for prospective studies. Second, the overall severity of disease in our study may generally be serious and 9 patients received liver transplantation during follow-up. In addition, we have not performed relevant verification in patients with other basic liver disease types, it will be interesting to study or test KP-AKI model in patients with cirrhosis but without ACLF.

In summary, almost 40% of patients with ACLF develop AKI. Among these, 25% had CA-AKI, which may benefit from frequent monitoring after discharge to improve outcomes. On the other hand, HA-AKI accounts for approximately 75% of AKI cases and can be avoided by eliminating its risk factors. Furthermore, this study developed a KP-AKI model for predicting AKI occurrence in ACLF patients and constructed an online calculator that is more convenient and accurate than the traditional scoring systems (CTP, MELD, and MELD-Na scores). The progression of AKI is common in patients with ACLF, and patients with a greater AKI and ICA-AKI stage progression were associated with worse prognoses among ACLF patients. Further studies are needed to validate our findings and to establish more effective prevention and treatment strategies to improve poor outcomes.

## Supplementary Information

**Additional file 1:** Comparison of clinical characteristics between training cohort and validation cohort.

## Data Availability

The clinical data in the study will not be shared publicly due to participants were informed at the time of providing consent that only researchers involved in the project would have access to the information they provided. But are available from the corresponding author on reasonable request.

## Abbreviations

ACLF: Acute-on-chronic liver failure
AKI: Acute kidney injury
AUROC: Receiver operator characteristic curve
ALB: Albumin
c-index: Concordance index
BUN: Blood urea nitrogen
CLD: Chronic liver disease
CA-AKI: Community-acquired AKI
eGFR: Estimated glomerular filtration rate
HA-AKI: Hospital-acquired acute kidney injury
HE: Hepatic encephalopathy
INR: International normalized ratio
MAP: Mean arterial pressure
PTs: Prothrombin time
sCr: Serum creatinine
TBIL: Total bilirubin
