# Supplementary material for: Characteristics of acute kidney injury and its impact on outcome in patients with acute-on-chronic liver failure
Source: BMC Gastroenterol. 2022 May 11;22:231. doi: 10.1186/s12876-022-02316-8 (PMC9092688; doi:10.1186/s12876-022-02316-8)
Supplement: Supplementary file 1 — Additional file 1: Comparison of clinical characteristics between training cohort and validation cohort. [file 12876_2022_2316_MOESM1_ESM.docx]

Table 1 Comparison of clinical characteristics between training cohort and validation cohort

| variables | Training cohort  (n=518) | Validation cohort  (n=174) | *P*-value |
| --- | --- | --- | --- |
| **Age (years)** | 50.8±12.2 | 43.6±10.5 | **<0.001** |
| **Male-n (%)** | 389(75.1) | 150(86.2) | **<0.001** |
| **Death-n(%)** | 164(31.7) | 36(20.7) | **0.020** |
| **AKI- n (%)** | 161(31.1) | 34(19.5) | **0.003** |
| **Concomitant diseases-n (%)** |  |  |  |
| Arterial hypertension | 73(14.1) | 15(8.6) | 0.061 |
| Diabetes mellitus | 89(17.2) | 12(6.9) | **<0.001** |
| **Etiology of liver disease-n (%)** |  |  | **<0.001** |
| Hepatitis B | 272(52.5) | 159(91.4) |  |
| Alcohol | 150(29.0) | 13(7.5) |  |
| Other causes | 96(18.5) | 2(1.1) |  |
| **Complication of liver disease-n (%)** |  |  |  |
| Ascites | 270(52.1) | 18(10.3) | **<0.001** |
| Encephalopathy | 47(9.1) | 21(20.4) | **<0.001** |
| GI bleeding | 95(18.3) | 2(1.1) | **<0.001** |
| Bacterial infection | 89(17.2) | 90(51.7) | **<0.001** |
| **Admission parameters** |  |  |  |
| MAP (mmHg) | 90.2±12.4 | 91.1±9.6 | 0.422 |
| Heart rate (bpm) | 83.6±13.9 | 90±61 | 0.172 |
| WBC (×10^9^/L) | 7.6±5.1 | 8.2±6.8 | 0.208 |
| PLT (×10^9^/L) | 100.1±63.6 | 112.2±53.1 | **0.026** |
| ALB(g/L) | 28.8±5.2 | 29.9±5.8 | **0.014** |
| TBIL(μmol/L) | 242.6±139.4 | 339.4±170.3 | **<0.001** |
| INR | 2.3±1 | 2.3±0.7 | 0.981 |
| PT(s) | 34.3±11.7 | 26.3±9.7 | **<0.001** |
| BUN(mmol/L) | 6.1±4 | 4.8±3 | **<0.001** |
| sCr (μmol/L) | 63.8±21.7 | 86.4±42.2 | **<0.001** |
| Serum Na^+^ (mmol/L) | 133.8±5.7 | 135±4.6 | **0.016** |
| Serum K^+^ (mmol/L) | 3.8±0.6 | 3.9±0.7 | 0.257 |
| CTP | 11.2±1.7 | 11.1±1.4 | 0.545 |
| MELD | 18.8±6.3 | 25±4.9 | **<0.001** |
| MELD-Na | 23.6±8.9 | 28±7.6 | **<0.001** |

*Estimated by MDRD. ALB: albumin; BUN, blood urea nitrogen; CTP, child-turcotte-pugh; eGFR, estimated glomerular filtration rate; HA-AKI, hospital-acquired acute kidney injury; HE, hepatic encephalopathy; INR: international normalized ratio; MAP, mean arterial pressure; MELD: model for end-stage liver disease; PLT: platelet;
